# A two-factor structure for cannabis use disorder identification test and its associations with demographic factors and cannabis use motives

**DOI:** 10.3389/adar.2026.16094

**Published:** 2026-03-02

**Authors:** Nadine Heckel, Carlos Nordt, Etna J. E. Engeli, Patricia Dürler, Marcus Herdener

**Affiliations:** Addictive Disorders Research Group, Department of Adult Psychiatry and Psychotherapy, Psychiatric University Hospital, University of Zurich, Zurich, Switzerland

**Keywords:** cannabis, confirmatory factor analysis, CUDIT-R, gender, motives, cannabis use disorder

## Abstract

**Introduction:**

Cannabis use is linked to the risk of developing a Cannabis Use Disorder (CUD), which can often be chronic. Early identification of problematic cannabis use is crucial to lower the risk of CUD and associated adverse effects. However, the factor structure of the widely used Cannabis Use Disorder Identification Test (CUDIT) remains ambiguous. Furthermore, the impact of age and gender on CUD assessed with CUDIT is unknown. Exploring cannabis use motives has been proposed to better understand susceptibility to CUD. This study aims to clarify the CUDIT’s factor structure, its links to cannabis use motives, and the influence of age and gender on CUD.

**Materials and Methods:**

We analyzed data from 3454 people who use cannabis (20.5% women; mean age = 30.31 years), collected from a Swiss online survey. Participants were categorized into four groups: younger men, younger women, older men, older women. Principal Component Analysis and Confirmatory Factor Analysis tested the factor structure of the revised CUDIT version (CUDIT-R). Structural Equation Modeling explored whether the influence of use motives on the CUDIT-R factors differs between demographic groups.

**Results:**

The results suggest that the CUDIT-R scale is best represented by two factors: Use Intensity (Cronbach’s α = 0.71) and Awareness of Problematic Use (Cronbach’s α = 0.72). Use Intensity was lowest for younger women, and younger participants were more aware of negative effects. Gender, age, and use motives uniquely relate with both CUDIT-R factors, highlighting the CUDIT-R’s potential to guide early identification and treatment of individuals at risk for CUD.

## Introduction

Despite its illegal status in many countries, the prevalence of cannabis use in Europe continues to rise [1, 2]. In Switzerland, where non-medical cannabis use is illegal, 5.5% of men and 2.5% of women aged 15–64 have used cannabis within the past month [3]. This raises concerns about possible public health issues as especially people with heavy cannabis use remain at risk for Cannabis Use Disorder (CUD) and cannabis-related negative consequences, such as anxiety, psychosis, and depression [4–6]. A review of the eleventh revision of the International Classification of Diseases (ICD-11) highlights that current treatment systems primarily target advanced stages of Substance Use Disorder (SUD) [7]. The authors propose a broader range of interventions for addressing problems related to substance use at an early stage. Implementing secondary prevention at this point can reduce the adverse health consequences of substance use and thus halt the development of SUD [8]. Identifying people with problematic cannabis use as early as possible is therefore of crucial importance. However, this poses a significant challenge, particularly due to the illegal status of cannabis, the limited awareness of associated risks among people who use cannabis (PWUC), and the diversity of PWUC.

There is a consensus on using the Cannabis Use Disorders Identification Test (CUDIT) to identify CUD risk in the past 6 months [9]. It assesses reasons for cannabis use, as well as patterns and consequences of use. The original 10-item CUDIT developed by Adamson and Sellman [10] showed good psychometric properties but this was only tested on a small sample of individuals with alcohol dependence (n = 53). To improve validity, the authors revised the scale using a clinical sample (n = 144). They identified a two-factor structure for the original CUDIT, reflecting two distinct but related underlying dimensions of the scale, and a one-factor structure for the new 8-item version [11]. A later study supported a one-factor structure for the new 8-item version, though it used a sample of college students with low cannabis use [12]. Notably, these studies all relied on small or highly specific samples. Annaheim et al. [13] revised the original 10-item CUDIT by replacing three poorly performing items, resulting in a 10-item CUDIT-R with improved psychometric properties in high-risk populations. They treated the scale as uni-dimensional but conducted factor analysis only before removing the poorly performing items. To strengthen the CUDIT’s validity and generalizability, it is essential to re-examine its factor structure using a large sample that includes participants across a broad range of CUDIT scores.

While the CUDIT is a reliable tool for identifying CUD, its lengthy and retrospective questions may not fit well in non-clinical questionnaires. Identifying simple, commonly assessed variables that relate to CUD can enable broad screening of all PWUC. These variables could streamline the CUDIT to people who may be at risk of CUD for further, more detailed assessment.

Past research has indicated the potential impact of demographic factors, like age and gender, on the risk of CUD. For example, men appear to have a higher prevalence of cannabis use and CUD compared to women, while women tend to transition more rapidly from initial use to CUD than men [12, 14, 15]. Cannabis use prevalence peaks in early to mid-twenties and declines afterwards with increasing age [1, 16]. Past research implies that rates of dependence are highest among younger adults [17] and younger PWUC may display more risky cannabis use patterns compared to older PWUC [18, 19]. However, older individuals remain susceptible to adverse effects associated with cannabis use. The prevalence of cannabis use among 35–64-year-olds increased by at least 50% between 2010 and 2019 [1]. This trend may be due to an aging population of PWUC or an increase in the medical use of cannabis especially among older PWUC [1, 20]. These differences between demographic groups highlight the need to further examine the specific relationships between age, gender, cannabis use, and the development of CUD.

Examining motives for cannabis use helps identify groups that are more susceptible to CUD as they have consistently been linked to the intensity and harms of cannabis use [21, 22]. Cannabis use as a coping mechanism for negative emotions has been linked to increased use frequency and adverse effects, including heightened distress [23–25]. Bonar et al. [26] found associations of coping motives, social motives, and using to enhance mood with higher use quantities. Furthermore, using cannabis as a coping mechanism, to enrich experiences, and for mood enhancement shows the strongest link to the risk of developing CUD, as assessed by the CUDIT [27].

Little is known, however, about how cannabis use motives relate to CUD across different demographic groups. One study found that younger patients receiving medical cannabis were more likely than older patients to endorse boredom as a motive for use [28]. It remains unclear whether this difference between age groups impacts the risk of CUD. While findings are mixed, gender also seems to influence use motives [23]. For example, coping motives have been found to be more strongly associated with cannabis use frequency in women than in men [29].

Building on these findings, the current study explores the factor structure of the CUDIT, and how demographic factors and use motives relate to problematic cannabis use (defined by the revised CUDIT scale by Annaheim et al. [13]). Moreover, it examines whether the relation between use motives and problematic use differs between demographic groups. This is a first step in improving the screening process and creating interventions for PWUC who have developed or might develop a CUD.

## Materials and methods

### Participants and procedures

Information was collected via an anonymous online survey (designed by the University of Zurich) in the German- and French-speaking parts of Switzerland from November-December 2016. Recruitment was conducted by several universities and city representatives, e.g., by regional newspapers, social media, and email newsletters. Participation required non-medical cannabis use within the past month and interest in participating in a future study investigating cannabis regulation. Of the 4334 participants who met these criteria, 3728 completed the questionnaire. To ensure single participation, respondents were asked if they had previously participated in the study. Based on this question, 145 participants were excluded. Of the remaining sample, 129 participants were excluded because they provided inconsistent information on their age or postcode. There was no age restriction for participation. The final sample consisted of 3454 PWUC who were between 10 and 99 years old. Because the survey was anonymous, ethical approval was not required.

### Measures

#### Demographic variables

Gender was coded as a binary variable (man/woman) and age was assessed as a continuous variable. Participants were categorized by age relative to the median ( ≤ 27 years = younger, >27 years = older), resulting in four demographic groups: younger men, younger women, older men, and older women.

#### Motives for cannabis use

Participants were asked to indicate the extent to which 22 motives for cannabis use applied to them on a 5-point Likert Scale (1 = “Never” to 5 = “Always”). 14 items were taken from the Marijuana Motives Measure [29], eight additional items from a study that had gathered motives from numerous sources [30]. The items were selected to cover a wide range of motives for using cannabis, while ensuring a concise questionnaire. To simplify the process, participants could rate as many items as they wanted. The mean for each motive was calculated to assess participants’ level of endorsement (Supplementary Table S1.).

#### Cannabis use disorder

For the present study, the revised 10-item CUDIT-R by Annaheim et al. [13] was used, which is based on data from the Swiss Cannabis Monitoring Study [31] (Supplementary Appendix S2). Throughout the manuscript, it is referred to as CUDIT-R. Most items used a 5-point Likert scale (0 = “Never” to 4 = “Daily or almost daily”). One question was altered to ask about use frequency over the previous 30 days instead of the previous 6 months (“Within the past 30 days, on how many days did you consume hashish/marijuana?”; The response options were 1 = “On 1–3 days”, 2 = “On 4–9 days”, 3 = “On 10–19 days”, 4 = “On 20 days or more”). As people without cannabis use in the previous 30 days were excluded from the study, this item adjustment is unlikely to meaningfully affect results. Using all items, a sum score ranging from 1–40 was calculated for each participant, with a higher score indicating higher risk of CUD. Annaheim et al. [13] do not suggest a cut-off score for CUDIT-R. Following previous suggestions, the cut-off scores 8 and 13 were compared [9].

### Data analysis

Data Analysis was conducted using R version 4.2.1 (2022-06-23 ucrt). First, Principal Component Analysis (PCA) with oblique rotation (oblimin) was performed on the ten CUDIT-R items to explore the factor structure of the CUDIT-R scale. Subsequently, based on the implications of PCA, a model for Confirmatory Factor Analysis (CFA) was developed to test whether the factor structure differed between four demographic groups. Moreover, it was explored whether motives for cannabis use were associated with CUDIT-R factors across demographic groups.

Factor analysis was justified by Bartlett’s test of sphericity ( χ 2 (45) = 8099.43, p < 0.001) confirming sufficient correlation between items for PCA [32, 33]. The Kaiser-Meyer-Olkin measure of sampling sphericity (KMO = 0.84, considered “great” [34]) verified sampling adequacy. ANOVA and Tukey’s HSD Test were used to compare the total CUDIT-R score and the CUDIT-R factors by demographic groups. Based on the implications of PCA, a model for CFA was developed. The analysis was completed using the R-package lavaan Version 0.6.12 and Maximum Likelihood estimation (ML). Since the χ 2 fit statistic is known to be overly sensitive with large sample sizes [35], the Comparative Fit Index (CFI), Root Mean Square Error of Approximation (RMSEA), and Standardized Root Mean Square Residual (SRMR) were considered for evaluating model fit. CFI >0.90, RMSEA <0.10, and SRMR <0.10 were considered to be an acceptable model fit [35].

To compare demographic groups, participants were split into two age categories based on the median age: younger ( ≤ 27 years) and older (>27 years). This created groups with an equal number of younger and older participants within each gender (Table 1). The age of 27 years also reflects a pragmatic cut-off that aligns with evidence on neurodevelopmental maturation, age-related declines in cannabis use prevalence, and the end of emerging adulthood [1, 36, 37]. Next, measurement invariance was tested by sequentially constraining parameters across the four demographic groups. Following Chen [38], metric invariance was evaluated with a −0.010 change in CFI, a 0.015 change in RMSEA, and a 0.030 change in SRMR. For scalar or residual invariance, a change in SRMR of 0.015 was considered sufficient [39]. To obtain a subset of use motives with expected high discriminatory power, all 22 motives were ranked according to their average rating, and the mid-50% motives were selected for further analysis (Supplementary Appendix S1). Motives endorsed by almost all or almost no participants show little variation and provide limited distinction between individuals or groups. Finally, a full path model was developed, adding the subset of ten use motives as predictors for CUDIT-R. Again, invariance was tested across the four demographic groups.

**TABLE 1:** Key figures of demographics and main variables.

Variable	Mean (SD)	Characteristics	Frequency (N = 3454)	Percent
Gender	​	Male	2,745	79.5%
​	​	Female	709	20.5%
Age	30.31 (11.63)	​	​	​
Use frequency (Past 30 days)	​	On 1–3 days	541	15.7%
​	​	On 4–9 days	609	17.6%
​	​	On 10–19 days	579	16.8%
​	​	On ≥ 20 days	1,725	49.9%
Demographic groups (Younger: ≤27 years, Older: >27 years)	​	Younger men	1,431	41.4%
​	​	Younger women	360	10.4%
​	​	Older women	349	10.1%
​	​	Older men	1,314	38.1%
CUDIT-R score (1–40)	11.18 (6.86)	Score ≥ 8	2,261	65.5%
​	​	Score ≥ 13	1,351	39.1%
CUDIT-R factors	​	​	​	​
Awareness of problematic use (0–24)	3.51 (3.85)	​	​	​
Use intensity (1–16)	7.67 (4.03)	​	​	​

SD, standard deviation.

## Results

### Demographic variables

Of the sample, 20.5% were women. Participants ranged in age from 10 to 99 years, with a mean age of 30.31 years (SD = 11.63; median = 27). Table 1 summarizes the demographics and other key variables.

### Factor structure of the CUDIT-R

#### Principal components analysis of CUDIT-R

The number of participants with heavy cannabis use was substantial for investigating CUD. The mean CUDIT-R score was 11.18 (SD = 6.86). 65.5% of participants were screened positive for probable CUD according to the 8-point cut-off and 39.1% according to the 13-point cut-off (Table 1). The CUDIT-R scale showed an acceptable value for Cronbach’s alpha ( α = 0.77). The correlation table for all ten CUDIT-R items can be found in Supplementary Appendix S2 (Supplementary Table S2.). PCA showed that two components, with eigenvalues over Kaiser’s criterion of 1, explained 50.1% of the variance. These components reflected Awareness of Problematic Use (Cronbach’s α = 0.72) and Use Intensity (Cronbach’s α = 0.71) (Table 2). The items “Difficulty in quitting cannabis use” and “Morning cannabis use for recovery after heavy use” loaded on both factors. The correlation between the two factors was statistically significant, yet small in the context of a factor analysis (r = 0.52, p < 0.001). This suggests that the two factors indeed reflect two different concepts. The scores for the two factors were calculated by summing up the values of the respective factor items with the scores for “Difficulty in quitting cannabis use” and “Morning cannabis use for recovery after heavy use” counting half towards each factor.

**TABLE 2:** Two components of the CUDIT-R scale as suggested by PCA.

CUDIT-R item	Oblique rotated factor loading	
	Awareness of problematic use	Use intensity
Failing to meet social expectations	**0.81**	−0.08
Difficulties at school or work	**0.77**	−0.14
Memory or concentration problems	**0.68**	0.16
Neglect of leisure activities	**0.61**	0.15
Concerns about cannabis use from others	**0.45**	0.11
Difficulty in quitting cannabis use	**0.37**	**0.49**
Morning cannabis use for recovery after heavy use	**0.33**	**0.35**
Use frequency	−0.07	**0.85**
Intoxication of more than 6 h	0.04	**0.74**
Reason for cannabis use (enjoyment or habit)	−0.01	**0.74**

Loadings over 0.30 are printed in bold.

ANOVA and Tukey’s HSD Test showed that when the CUDIT-R score was taken as a one-factor concept, younger men ( ≤ 27 years) had a significantly higher CUDIT-R score than all other demographic groups (Table 3). Looking at CUDIT-R as a two-factor concept gave a more nuanced picture of the group differences. ANOVA showed that for both factors, there was a significant difference between at least two of the four demographic groups. Results from the Tukey’s HSD Test showed that younger men and women were significantly more aware of the negative effects of their cannabis use than older participants. At the same time, young women scored significantly lower on the Use Intensity scale than the other groups.

**TABLE 3:** CUDIT-R: ANOVA with post-hoc pairwise comparison of demographic groups.

CUDIT-R total score				
ANOVA	F (3, 3450) = 5.63 p < 0.001			
Mean values	Group	Mean	SD	
	Younger men	11.70	6.89	
	Younger women	10.60	7.46	
	Older men	11.00	6.75	
	Older women	10.35	6.39	
Pairwise comparison	Compared groups	Diff	CI 95%	p
	Younger men - younger women	1.10	[0.06; 2.14]	0.033
	Younger men - older men	0.70	[0.03; 1.37]	0.037
	Younger men - older women	1.35	[0.30; 2.40]	0.005
	Younger women - older women	0.25	[-1.07; 1.58]	0.961
	Older men - younger women	0.40	[-0.64; 1.45]	0.763
	Older men - older women	0.65	[-0.41; 1.71]	0.391

CUDIT-R factors				
Awareness				
ANOVA	F (3, 3450) = 26.0 p < 0.001			
Mean values	Group	Mean	SD	
	Younger men	4.12	4.02	
	Younger women	3.73	4.12	
	Older men	3.04	3.61	
	Older women	2.60	3.26	
Pairwise comparison	Compared groups	Diff	CI 95%	p
	Younger men - younger women	0.39	[-0.19; 0.97]	0.305
	Younger men - older men	1.08	[0.71; 1.45]	<0.001
	Younger men - older women	1.52	[0.94; 2.11]	<0.001
	Younger women - older women	1.13	[0.40; 1.87]	<0.001
	Older men - younger women	0.69	[-1.27; −0.11]	0.012
	Older men - older women	0.44	[-0.15; 1.03]	0.216

Use intensity				
ANOVA	F (3, 3450) = 7.29 p < 0.001			
Mean values	Group	Mean	SD	
	Younger men	7.58	3.90	
	Younger women	6.87	4.15	
	Older men	7.96	4.09	
	Older women	7.75	4.12	
Pairwise comparison	Compared groups	Diff	CI 95%	p
	Younger men - younger women	0.71	[0.10; 1.32]	0.015
	Younger men - older men	−0.38	[-0.77; 0.02]	0.065
	Younger men - older women	−0.17	[-0.79; 0.45]	0.893
	Younger women - older women	−0.88	[-1.66; −0.10]	0.019
	Older men - younger women	1.08	[0.47; 1.70]	<0.001
	Older men - older women	0.21	[-0.41; 0.83]	0.824

N = 3454; SD = standard deviation; Demographic groups are split at the median age (younger: ≤ 27 years, older: >27 years); Pairwise comparisons was conducted using Tukey’s HSD test for multiple comparisons of means; Diff = difference of the mean score of the compared groups; CI 95% = the 95% family-wise confidence level; p = adjusted value for significance.

#### CUDIT-R measurement model: CFA

**FIGURE 1:**
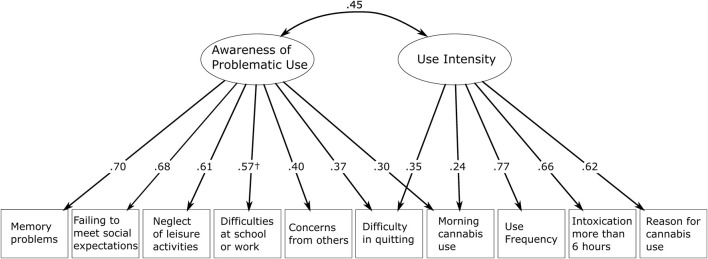
Measurement model of CUDIT-R scale with dual-factor composition and standardized factor loadings. ^†^Standardized loadings for “Difficulties at school or work” on Awareness of Problematic Use were released to differ between groups: It was higher for younger women (standardized coefficient = 0.62) than for younger men (0.59), older men (0.55), and older women (0.21). Demographic groups are split at the median age (younger: ≤ 27 years, older: >27 years). The figure shows the measurement model of CUDIT-R with assumed partial invariance (Model fit: CFI = 0.940, RMSEA = 0.060, SRMR = 0.046). All standardized factor loadings are highly significant (p < 0.001).

Based on the results from PCA, a measurement model of the CUDIT-R scale was developed using CFA to compare the factor structure across demographic groups. Awareness of Problematic Use and Use Intensity were set as latent factors with the ten manifest CUDIT-R items loading onto them (Figure 1). The CFA produced a large and significant χ 2 value, however, this might have resulted mainly from the large sample size. Yet, CFI, RMSEA, and SRMR confirmed an acceptable model fit (Supplementary Table S3.). All factor loadings were positive and highly significant. To test measurement invariance, an unconstrained (configural) model was established across the four groups. It showed an acceptable model fit. Next, factor loadings were restricted to be equal across the four demographic groups (metric invariance model), which led to a meaningful decrease in model fit, indicating that full metric invariance did not hold and factor loadings differed across groups. Releasing constraints on loadings individually showed that when permitting the factor loading of the “Difficulties at school or work” item to vary between groups, the fit indices closely approximated those of the unconstrained model. Releasing constraints on other loadings did not yield sufficient improvements. Given this partial metric invariance, all loadings are comparable between groups, except for the loading of the “Difficulties at school or work” item on Awareness of Problematic Use which was highest for younger women. When comparing only gender or only age groups, factor loadings were invariant between men and women and between younger and older participants, meaning that full metric invariance was achieved and indicating stronger invariance in these simpler splits.

### Structural model: CUDIT-R factors and motives

**FIGURE 2:**
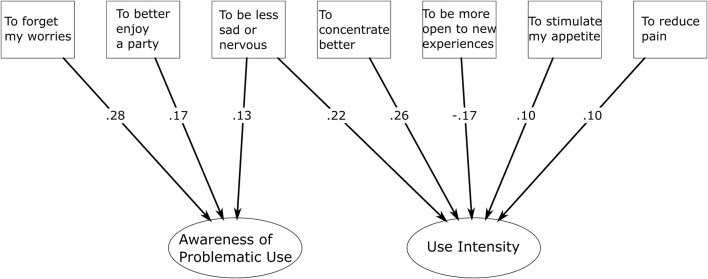
Structural model - Dual-factor composition of the CUDIT-R with cannabis use motives as predictors. The Figure shows a subset of use motives and their standardized regression coefficients on the CUDIT-R factors (structural model). Due to model complexity, simplifications were made: The measurement model is not included. It closely resembles the one displayed in Figure 1. Significance levels are not included. All displayed standardized regression weights are highly significant (p < 0.001). Insignificant regression coefficients and coefficients ≤ 0.10 were omitted for clarity. Consequently, the use motives “to be more sociable”, “to improve my sexuality”, and “to be more creative” are not included. The latter had a significant but very small negative effect on Awareness of Problematic Use. The complete path model can be found in Supplementary Appendix S3 (Supplementary Table S4.).

To obtain the full path model, the subset of mid-50% motives were added to the model as manifest predictors of the two CUDIT-R factors (structural model; Figure 2). The model fit was acceptable as was the model fit for the unconstrained model that compared the four demographic groups. Testing for measurement invariance replicated the findings of the previous CFA, suggesting a partial metric invariance with the factor loadings being restricted to equality, and only the factor loading for “Difficulties at school or work” on Awareness of Problematic Use allowed to vary between groups. Restricting the regression coefficients of this partial metric invariance model to equality did not meaningfully decrease model fit, suggesting that these coefficients were equal across groups. Using cannabis to forget worries, reduce sadness or nervousness, alleviate pain, improve appetite, and to enhance a party experience was positively related to one or both CUDIT-R factors. In contrast, using cannabis for higher creativity and openness to new experiences was linked to lower Awareness of Problematic Use or lower Use Intensity. Using cannabis for better concentration was associated with higher awareness but lower Use Intensity. Although some of these connections were relatively weak, use motives explained approximately 17.3% of the variance in awareness and 25.5% of the variance in intensity. Detailed results can be found in Supplementary Appendix S3 (Supplementary Table S4.).

## Discussion

In the light of increasing lifetime prevalence of cannabis use and as emphasized in a review of the ICD-11, early identification of problematic substance use is crucial to implementing effective secondary prevention measures [7]. This can potentially mitigate adverse health consequences and prevent the progression to more severe stages of SUD.

Our findings suggest that understanding CUD is improved by using a two-factor solution for the CUDIT-R scale. This contrasts with earlier studies that have treated the CUDIT as a uni-dimensional construct [11–13]. However, these studies examined different versions of the CUDIT and relied on small or highly specific samples. The present study can therefore add value by examining the CUDIT using a larger sample with a broader range of CUDIT scores.

The two CUDIT-R factors can help identify meaningful subgroups among PWUC and approach them appropriately. The factor Use Intensity captures the frequency and intensity of cannabis use, with higher scores indicating more frequent use, extended intoxication (>6 h), and habitual use. The factor Awareness of Problematic Use reflects the negative effects of cannabis use on personal, social, and work life, and awareness of these issues. This factor also encompasses concerns expressed by people around the person who uses cannabis. PWUC with a high Awareness of Problematic Use may accept direct help and personalized interventions more readily. Conversely, PWUC with low awareness might be less receptive, necessitating efforts to increase their awareness of risks of cannabis use. PWUC with high Use Intensity may profit from interventions focused on reducing use, regaining control, and learning safer-use practices.

Our results highlight the potential of using simple and commonly assessed variables-such as age and gender-to screen all PWUC efficiently and identify those who may be at risk of CUD. Previous findings suggest that prevalence of cannabis use and CUD is higher among men than women [12, 14, 15] and that cannabis use prevalence, rates of cannabis dependence, and risky cannabis use seem to be higher among younger participants [1, 16–18]. Our findings allow a more nuanced interpretation. CFA suggested that in our sample, younger men and women ( ≤ 27 years) did not show significantly higher Use Intensity than older men and older women. In fact, younger women have a significantly lower Use Intensity than other demographic groups. At the same time, younger women and men are more burdened by negative effects than older participants. This could be due to challenges in social and working life that this demographic group commonly faces, such as frequent changes in relationships and employment [36]. In our sample, especially for younger women, challenges in school and work settings seem to have a particular strong relation to perceived negative effects of cannabis use. This suggests that although this group shows a significantly lower use intensity and may therefore be seen as needing fewer preventive or treatment measures, it may still require specific attention. Evidence also shows that neurological effects of regular or heavy cannabis use, such as cognitive deficits in attention, learning, and memory, are more severe and persistent in younger PWUC [6, 40]. Meanwhile, older PWUC may suffer less from adverse neurological effects, have more stable social and work lives, and have learned to avoid negative effects or integrate them into their lives. Alternatively, those aware of negative effects may cease usage, while unaware PWUC continue into older age.

Our findings also provide a detailed understanding of the relationship between cannabis use motives and CUD. Participants who used cannabis to cope with worries, sadness, or nervousness, or to enhance party experiences, were more aware of the negative effects. Using cannabis to alleviate sadness or nervousness was additionally related to higher Use Intensity. The observed association between coping related cannabis use motives and increased use intensity and adverse effects is consistent with previous findings showing that coping motives are reliably linked to more frequent use and greater negative consequences [23–25]. Participants using cannabis for self-medication (appetite stimulation, pain reduction) or enhanced concentration showed more intensive use but no heightened awareness of cannabis-related problems. These effects were consistent across demographic groups. Personalized interventions can therefore be improved by targeting cannabis use motives [26]. Offering alternatives for addressing PWUC’ motives could reduce negative effects and use intensity. For example, PWUC endorsing coping motives could learn alternative ways to manage worries, sadness, and nervousness. For those using cannabis to increase concentration, it may be beneficial to raise awareness that cannabis does not enhance concentration and may impair cognitive performance [6, 41].

In conclusion, motives for cannabis use, age, and gender which are brief items commonly implemented in (non-clinical) questionnaires, are associated with the two factors of CUDIT-R that we established here. These variables could support early identification of CUD by enabling efficient, broad screening of all PWUC to identify those with potential problematic cannabis use. These individuals could then undergo detailed assessment using longer tools like the CUDIT. Moreover, interventions should be tailored to consider PWUC’s motives for use and demographic background. Such a personalized approach may improve outcomes in the prevention and treatment of CUD.

### Limitations

Our study benefits from a large sample size with substantial variance in CUDIT-R scores. To reach such a high number of PWUC, snowball-sampling was used. The survey was directed at German and French speakers in Switzerland with past-month cannabis use and interest in a pilot study with cannabis. Therefore, this sample is not representative of PWUC in Switzerland.

Another limitation is that due to the cross-sectional nature of the data, causal relationships between variables and changes over time cannot be explored. For multigroup-comparison, participants were categorized into two broad age groups (18–27 and 28–80 years), simplifying analyses but limiting the possibility to capture nuanced age effects and potential non-linear relationships within groups. Lastly, despite the anonymity of our online questionnaire, social desirability bias may still affect the data.

### Future research

Future research should consider the CUDIT-R as a two-factor concept. For the score calculation of these two concepts, age and gender differences should be taken into account. For example, a higher weight could be assigned to problems at school or work for younger women when calculating the score for Awareness of Problematic Use. Future longitudinal studies can enhance understanding of the interplay between cannabis use motives, demographic factors, and CUD development. Exploring smaller age categories can capture more variations in use motives and CUD across different age groups.

While gender, age, and use motives offer insights into identifying individuals at risk of CUD early on, they account for a small portion of the variance in CUDIT-R factors. Hence, future research should explore additional aspects, such as education, employment status, or social environment that could be linked to CUD.

## Conclusion

Addressing the need for better early identification of and personalized interventions for problematic substance use, our study explored the CUDIT-R scale by Annaheim et al. [13] and its relation to age, gender, and cannabis use motives. Our results propose a two-factor solution for the CUDIT-R scale with the underlying factors Use Intensity and Awareness of Problematic Use. In our sample, Use Intensity was lowest for younger women, and younger participants were more aware of the negative effects of their use. Higher endorsement of the use motives ”to forget my worries”, ”to be less sad or nervous”, and ”to better enjoy a party” was related to both higher Use Intensity and Awareness of Problematic Use across demographic groups. Therefore, motives for cannabis use, age, and gender could help with the early identification of individuals at risk for CUD. These findings should also inform the design of tailored interventions for people with problematic use of cannabis.

## Data Availability

The datasets presented in this article are not readily available because because data sharing is not applicable. Requests to access the datasets should be directed to nadine.heckel@bli.uzh.ch.
