# Supplementary material for: A two-factor structure for cannabis use disorder identification test and its associations with demographic factors and cannabis use motives
Source: Adv Drug Alcohol Res. 2026 Mar 2;6:16094. doi: 10.3389/adar.2026.16094 (PMC12989445; doi:10.3389/adar.2026.16094)
Supplement: Supplementary file 1 [file Supplementaryfile1.pdf]

Supplementary Material

1 CANNABIS USE MOTIVES

Table S1 shows all 22 cannabis use motives presented to participants in the online questionnaire, ranked according to their mean value in descending order. The question was "There are various reasons for using cannabis. Which of the following statements applies to you?". Responses were given on a 5-point Likert-scale (1 = "Never", 2 = "Rarely", 3 = "Sometimes", 4 = "Often", 5 = "Always"). Participants could rate as many motives as they wanted. The motives were translated to German with necessary adaptations to the original wording.

To select motives that could most likely detect differences between participants, the mid-50% motives according to endorsement were chosen. They likely have higher discriminatory potential than motives endorsed by almost all or almost no participants. The top 25% and bottom 25% of motives according to endorsement show little variation and do not allow meaningful distinctions between individuals or groups. For example, although using cannabis due to peer pressure may have a strong association with CUDIT-R factors, only 14 participants reported using cannabis often or always for this reason.

To validate our choice, we conducted a sensitivity analysis by repeating the SEM analysis of the full structural model including all 22 cannabis use motives. This model showed insufficient fit according to our defined criteria (CFI = 0.885, RMSEA = 0.041, SRMR = 0.029).

2 CUDIT-R VERSIONS AND CORRELATION MATRIX FOR CUDIT-R ITEMS

For the present study, we used the 10-item CUDIT-R version by Annaheim et al. (2010) which builds on the original CUDIT version by Adamson and Sellman (2003). It replaced three poorly performing items with new ones and, as a result, the scale showed better psychometric performance for PWUC with a high problem score. For developing the CUDIT-R scale, Annaheim et al. (2010) regarded the CUDIT scale as a one-factor concept. However, they only tested the factor structure before excluding three items. The CUDIT-R assesses for example PWUC' reasons for using cannabis (e.g. "How often during the past six months did you need to use cannabis in the morning to get yourself going after a heavy session of using cannabis?") and the impact that their use has on their lives (e.g. "How often during the past six months did you fail to do what was normally expected from you because of using cannabis?"). For most items, participants selected their response from a 5-point Likert-Scale (0 = "Never", 1 = "Less than monthly", 2 = "Monthly", 3 = "Weekly", 4 = "Daily or almost daily"). For two questions (e.g. "Has a relative, friend or a doctor, or other health worker been concerned about your use of cannabis over the past six months?") with only two response options, responses were coded so that the response indicating a more problematic use (in this example: "yes") was assigned the value 4 and the other response (in this example: "no") the value 0. Table S2 shows the ten CUDIT-R items and their correlations.

Table S1. Cannabis use motives with key figures

Motive	Mean	SD	N
Because I like the feeling†	4.24	0.82	3426
Because it gives me a pleasant feeling†	3.93	0.91	3373
Because it's fun†	3.82	1.09	3369
To get high†	3.59	1.19	3381
To help me get to sleep at night‡	3.41	1.29	3370
Because it makes social gatherings more fun†	3.21	1.16	3351
Because it helps me be more creative and original†	2.85	1.25	3342
Because it helps me when I feel depressed or nervous†	2.57	1.31	3326
I use cannabis to help with pain symptoms‡	2.49	1.25	3366
Because it helps me enjoy a party†	2.39	1.20	3331
To be more open to experiences†	2.33	1.25	3291
To improve concentration‡	2.28	1.26	3304
To forget my worries†	2.23	1.22	3329
To be sociable†	2.09	1.11	3328
To heighten sexual experiences‡	1.96	1.11	3331
To improve my appetite‡	1.93	1.14	3293
Because I feel more self-confident and sure of myself†	1.87	1.08	3319
To control muscle spasms‡	1.81	1.20	3331
To reduce symptoms of nausea‡	1.77	1.14	3300
To help reduce anxious feelings‡	1.76	1.10	3320
Because my friends pressure me†	1.11	0.41	3278
So I won't feel left out†	1.09	0.40	3337

†Use Motives taken from Simons et al. (1998); ‡Use Motives taken from McDonald (2020). Mean = average rating of motive endorsement, the motives are ranked according to this value in descending order; SD = Standard Deviation; N = number of participants who rated the respective motive (total N = 3454). The box contains the mid-50% motives that were used for the structural model.

Table S2. Correlation matrix for CUDIT-R items

	Correlations									
Variables	(1)	(2)	(3)	(4)	(5)	(6)	(7)	(8)	(9)	(10)
(1) Failing to meet social expectations	-									
(2) Difficulties at school or work	0.46	-								
(3) Memory or concentration problems	0.47	0.38	-							
(4) Neglect of leisure activities	0.42	0.35	0.41	-						
(5) Concerns about cannabis use from others	0.26	0.21	0.28	0.21	-					
(6) Difficulty in quitting cannabis use	0.31	0.24	0.40	0.34	0.29	-				
(7) Morning cannabis use for recovery	0.26	0.22	0.31	0.21	0.20	0.31	-			
(8) Use Frequency	0.13	0.10	0.27	0.25	0.16	0.36	0.24	-		
(9) Intoxication of more than 6 hours	0.19	0.15	0.27	0.24	0.17	0.34	0.34	0.53	-	
(10) Reason for cannabis use (enjoyment/habit)	0.16	0.13	0.23	0.25	0.14	0.38	0.21	0.50	0.34	-

N = 3454; All displayed correlations are highly significant.

3 RESULTS FOR MODEL TESTING

Results for model fit indices

Table S3 shows the model fits for the measurement model and structural model for CUDIT-R. Testing invariance between the four demographic groups for the measurement model showed that the loading of the ”Difficulties at school or work” item on Awareness of Problematic Use was highest for younger women. To determine whether this difference stemmed from a difference between genders or age categories, an unconstrained (configural) model was fitted to compare genders which showed acceptable model fit. Restricting the factor loadings to be equal for men and women (metric model) did not decrease model fit meaningfully. Similarly, a configural model comparing age categories also showed acceptable fit, and restricting factor loadings to be equal between age categories did not meaningfully decrease model fit. These results suggest that the factor loadings were invariant between genders and age categories.

Structural Equation Modeling

Table S4 shows the detailed results of the Structural Equation Modeling analysis.

REFERENCES

Simons J, Correia CJ, Carey KB, Borsari BE. Validating a five-factor marijuana motives measure: Relations with use, problems, and alcohol motives. *J Couns Psychol* **45** (1998) 265–273. doi:10.1037//0022-0167.45.3.265.

McDonald C. Identifying cannabis use motives and their association with problematic cannabis use [Master’s thesis, The University of New Brunswick] (2020). Accessed on April, 7, 2025.

Annaheim B, Scotto TJ, Gmel G. Revising the Cannabis Use Disorders Identification Test (CUDIT) by means of Item Response Theory. *Int J Methods Psychiatr Res* **19** (2010) 142–155. doi:10.1002/mpr.308.

Adamson SJ, Sellman JD. A prototype screening instrument for cannabis use disorder: The Cannabis Use Disorders Identification Test (CUDIT) in an alcohol-dependent clinical sample. *Drug Alcohol Rev* **22** (2003) 309–315. doi:10.1080/0959523031000154454.

Table S3. Measurement invariance testing: Model fits for all models

		Model fit			
		$\chi2$(df)	CFI	RMSEA(90% CI)	SRMR
CUDIT-R Measurement model		485.87(32)	.944	.064(.059-.069)	.038
	Groups[†]				
	Younger men (n = 1431)	208.66(32)	0.949	0.062(0.054-0.070)	0.038
	Younger women (n = 360)	85.93(32)	0.948	0.068(0.051-0.086)	0.043
	Older men (n = 1314)	200.74(32)	0.942	0.063(0.055-0.072)	0.039
	Older women (n = 349)	80.57(32)	0.932	0.066(0.048-0.084)	0.047
	Invariance testing				
	Four demographic groups				
	Configural model	575.89(128)	0.945	0.064(0.058-0.069)	0.040
	Metric model	851.48(158)	0.915	0.071(0.067-0.076)	0.059
	Partial model	642.06(155)	0.940	0.060(0.056-0.065)	0.046
	Genders				
	Configural model	499.73(64)	0.946	0.063(0.058-0.068)	0.038
	Metric model	554.88(74)	0.941	0.061(0.057-0.066)	0.043
	Age Categories				
	Configural model	507.40(64)	0.945	0.063(0.058-0.069)	0.038
	Metric model	587.09(74)	0.936	0.063(0.059-0.068)	0.046
Full SEM model		977.16(112)	0.905	0.049(0.046-0.052)	0.034
	Invariance testing				
	Configural	1276.86(448)	0.909	0.048(0.045-0.051)	0.036
	Metric	1545.93(478)	0.883	0.053(0.050-0.056)	0.045
	Partial	1340.60(475)	0.905	0.048(0.045-0.051)	0.038
	Restricted Regression Weights	1416.99(535)	0.903	0.045(0.043-0.048)	0.041

[†]Demographic groups are split at the median age (younger: $\leq$ 27 years, older: $>$27 years). $\chi2$ = chi-square difference statistic; df = degrees of freedom; CFI = Comparative Fit Index; RMSEA = Root Mean Square Error of Approximation; 90% CI = 90% Confidence Intervall of the RMSEA; SRMR = Standardized Root Mean Square Residual; The full SEM model includes the measurement model for CUDIT-R (Figure 1 in the main manuscript) and the structural model including a subset of use motives as predictors (Figure 2 in the main manuscript).

Table S4. Full SEM model: loadings and regression weights per demographic group

	Younger men		Older men		Older women		Younger women	
**Measurement model: Loadings of CUDIT-R items on latent variables (CUDIT-R factors)**								
	Estimate	p	Estimate	p	Estimate	p	Estimate	p
**Awareness of Problematic Use**								
Memory Problems	0.70		0.71		0.73		0.72	
Failing to meet social expectations	0.65	<0.001	0.67	<0.001	0.71	<0.001	0.68	<0.001
Neglect of Leisure Activities	0.62	<0.001	0.58	<0.001	0.64	<0.001	0.68	<0.001
Difficulties at school or work[1]	0.58	<0.001	0.54	<0.001	0.22	<0.001	0.62	<0.001
Concerns from others	0.37	<0.001	0.40	<0.001	0.44	<0.001	0.41	<0.001
Difficulty in quitting	0.43	<0.001	0.37	<0.001	0.34	<0.001	0.44	<0.001
Morning cannabis use	0.26	<0.001	0.23	<0.001	0.27	<0.001	0.29	<0.001
**Use Intensity**								
Difficulty in quitting	0.33		0.33		0.29		0.33	
Morning cannabis use	0.28	<0.001	0.29	<0.001	0.33	<0.001	0.29	<0.001
Use frequency	0.77	<0.001	0.77	<0.001	0.73	<0.001	0.75	<0.001
Intoxication >6 hours	0.72	<0.001	0.65	<0.001	0.60	<0.001	0.68	<0.001
Reason for cannabis use	0.61	<0.001	0.62	<0.001	0.62	<0.001	0.65	<0.001
**Structural model: Regression weights of use motives on CUDIT-R factors**								
	Estimate	p	Estimate	p	Estimate	p	Estimate	p
**Awareness of Problematic Use**								
To be more creative	-0.05	0.029	-0.05	0.029	-0.06	0.029	-0.05	0.029
To be less sad or nervous	0.13	<0.001	0.14	<0.001	0.15	<0.001	0.12	<0.001
To reduce pain	-0.03	0.187	-0.03	0.187	-0.03	0.187	-0.03	0.187
To better enjoy a party	0.14	<0.001	0.15	<0.001	0.14	<0.001	0.13	<0.001
To be more sociable	-0.01	0.817	-0.01	0.817	-0.01	0.817	-0.01	0.817
To improve my sexuality	0.02	0.326	0.02	0.326	0.02	0.326	0.02	0.326
To stimulate my appetite	0.03	0.092	0.04	0.092	0.04	0.092	0.03	0.092
To concentrate better	-0.07	0.002	-0.07	0.002	-0.08	0.002	-0.06	0.002
To forget my worries	0.28	<0.001	0.28	<0.001	0.29	<0.001	0.27	<0.001
To be more open to new experiences	0.01	0.811	0.01	0.811	0.01	0.811	0.01	0.811
**Use Intensity**								
To be more creative	-0.02	0.363	-0.02	0.363	-0.02	0.363	-0.02	0.363
To be less sad or nervous	0.22	<0.001	0.21	<0.001	0.23	<0.001	0.21	<0.001
To reduce pain	0.10	<0.001	0.10	<0.001	0.11	<0.001	0.10	<0.001
To better enjoy a party	0.09	<0.001	0.08	<0.001	0.08	<0.001	0.08	<0.001
To be more sociable	0.00	0.850	0.00	0.850	0.00	0.850	0.00	0.850
To improve my sexuality	-0.01	0.713	-0.01	0.713	-0.01	0.713	-0.01	0.713
To stimulate my appetite	0.11	<0.001	0.10	<0.001	0.11	<0.001	0.10	<0.001
To concentrate better	0.26	<0.001	0.25	<0.001	0.26	<0.001	0.25	<0.001
To forget my worries	0.10	<0.001	0.09	<0.001	0.09	<0.001	0.10	<0.001
To be more open to new experiences	-0.17	<0.001	-0.15	<0.001	-0.15	<0.001	-0.16	<0.001
**Covariance**								
	Estimate	p	Estimate	p	Estimate	p	Estimate	p
Awareness of Problematic Use & Use Intensty	0.47	<0.001	0.44	<0.001	0.45	<0.001	0.59	<0.001

[1] Standardized loadings for ”Difficulties at school or work” on Awareness of Problematic Use were released to differ between demographic groups.

Estimates = standardized estimates for loadings and regression weights; p = significance level

The table shows loadings and regression weights of the full SEM model (including the measurement and structural models) for each demographic group. All loadings and regression weights are restricted to be equal across groups, except for ”Difficulties at school or work”. Estimates differ slightly due to different group sizes.
